# Assessment of Intraspecific Diversity and Screening of Elite Genotypes of *Atriplex canescens* as a Host Plant for *Cistanche deserticola*

**DOI:** 10.3390/plants15060881

**Published:** 2026-03-12

**Authors:** Qingyun Pang, Minghao Huang, Lingcong Xu, Liang Shen, Fan Wang, Jianjun Qi, Rong Xu, Changqing Xu

**Affiliations:** 1 State Key Laboratory for Quality Ensurance and Sustainable Use of Dao-Di Herbs, Institute of Medicinal Plant Development, Chinese Academy of Medical Sciences & Peking Union Medical College, Beijing 100193, China; s2023009039@student.pumc.edu.cn (Q.P.); s2025009038@student.pumc.edu.cn (M.H.); s2024009038@student.pumc.edu.cn (L.X.); fwang@implad.ac.cn (F.W.); jjqi@implad.ac.cn (J.Q.); cqxu@implad.ac.cn (C.X.); 2 Natural History Museum of China, Beijing 100050, China

**Keywords:** *Cistanche deserticola*, *Atriplex canescens*, germplasm evaluation, ITS, integrated assessment, genetic diversity

## Abstract

Screening superior hosts is critical for artificial cultivation of *Cistanche deserticola* Y.C.Ma. However, intraspecific trait variation and host suitability of its emerging host *Atriplex canescens* (Pursh) Nutt remain unsystematically evaluated. In this study, 31 *A. canescens* individuals with diverse morphotypes and parasitic statuses were selected from over 300 seedlings. After post-transplant assessment, 17 representative *A. canescens* genotypes were selected and cutting-propagated. Their genetic, phenotypic and photosynthetic traits were systematically analyzed by multiple approaches, with a multi-indicator evaluation system built by correlation analysis and entropy-weighted Technique for Order Preference by Similarity to Ideal Solution)(TOPSIS). The results showed that the Internal Transcribed Spacer(ITS) sequences of the selected genotypes had an approximate length of 644 bp, exhibiting an average GC contents of 58.35%. A total of 22 haplotypes were detected, indicating high genetic diversity. In this study, superior host genotypes were defined as those with relatively excellent growth potential and stable, efficient photosynthetic performance. NP3.13, P3.1 and NP2.23 were recognized as promising candidate host genotypes with potential for *C. deserticola* cultivation, and their host suitability was indirectly inferred from their relatively superior growth and photosynthetic traits. This study not only provides valuable candidate germplasm resources and a scientific basis for optimizing the cultivation of *C. deserticola*, but also furnishes methodological support for elite genotypes screening in other plant species by the established evaluation framework.

## 1. Introduction

*Cistanche deserticola* Y. C. Ma is a holoparasitic plant belonging to the family Orobanchaceae. Endemic to the desert regions of northwestern China, this species is a highly valuable medicinal herb renowned for its unique medicinal, edible, and economic values. *C. deserticola* primarily parasitizes the roots of host plants including *Atriplex canescens* (Pursh) Nutt. and *Haloxylon ammodendron* [1]. *A. canescens*, a species of the family Chenopodiaceae, is indigenous to the Midwestern United States and was introduced to northwestern China in the 1990s. This semi-evergreen shrub is widely utilized for windbreak construction, sand fixation, pasture development, and saline-alkali land reclamation, exhibiting strong stress tolerance and broad adaptability [2]. Recent studies have demonstrated that *Cistanche* parasitizing *A. canescens* exhibits significantly higher levels of key medicinal components (e.g., phenethyl alcohol glycosides and polysaccharides) as well as higher medicinal yields compared to those parasitizing *H. ammodendron*, indicating substantial application potential [3].

With the rapid decline of wild *C. deserticola* resources, artificial cultivation has become indispensable for ensuring the sustainable development of the *Cistanche* industry. Improving the parasitism rate and yield of *C. deserticola* is critical for the success of artificial cultivation. Consequently, the host plant species and its physiological growth status significantly affect the parasitism rate and medicinal yield of *C. deserticola* [4]. Previous studies have indicated that significant morphological and physiological variations exist among different *A. canescens* genotypes within the same habitat [5,6]. Similarly, the parasitism of *C. deserticola* varies among host individuals or genotypes: Some *A. canescens* hosts are successfully colonized, while others remain uncolonized or exhibit only mild parasitism, consistent with the relationship between host variation and parasitism efficacy of *C. deserticola* on its typical host *H. ammodendron* [7,8]. These variations in parasitic status may be correlated with differences in host growth vigor and photosynthetic efficiency, which in turn influence the medicinal yield of *C. deserticola* [9,10].

However, most existing studies on *A. canescens* have primarily focused on its stress resistance mechanisms [11,12,13], ecological applications, and adaptability to new environments [14,15,16]. Fundamental research on the germplasm resources of *A. canescens* as a host for *C. deserticola* remains relatively underdeveloped, particularly regarding the systematic evaluation of phenotypic traits and growth responses to parasitic stress among different genotypes. This research gap has, to a certain extent, hindered the targeted screening of high-potential host genotypes. Comprehensive germplasm evaluation serves as the cornerstone of genetic research and breeding practices [17,18]. Therefore, establishing an integrated evaluation system that combines phenotypic, physiological, and genetic data is critical for screening host germplasm with both stress resistance and high-yield potential [19,20,21]. To achieve the quantitative evaluation and ranking of host genotypes, this study adopts the TOPSIS (Technique for Order Preference by Similarity to an Ideal Solution) multi-criteria decision-making model, which has been successfully applied in crop variety screening [22,23,24,25]. This model can comprehensively consider agronomic, economic, and quality traits, thereby facilitating the identification of high-yielding varieties with good marketability [26,27]. It provides the valuable methodological support for the systematic screening of high-potential *C. deserticola* hostgenotypes in this study.

In this study, *A. canescens* genotypes with variations in *C. deserticola* parasitism rates and post-inoculation growth characteristics were selected. By measuring agronomic traits, leaf morphological characteristics, and chlorophyll fluorescence parameters, combined with ITS sequence analysis to determine the genetic background and phylogenetic relationships among genotypes, we clarified their genetic diversity and haplotype distribution patterns. This approach enables the elucidation of genetic, phenotypic, and physiological variations among genotypes, as well as their suitability as hosts for *C. deserticola*. Furthermore, a multi-indicator evaluation system was established by using Pearson correlation analysis and an entropy-weighted TOPSIS model to identify superior host genotypes. This study provides a scientific basis for host selection, optimization of artificial cultivation models, and the high-quality development of the *C. deserticola* industry.

## 2. Results

### 2.1. Genetic Diversity Analysis of A. canescens Genotypes Based on ITS Sequences

#### 2.1.1. Basic Characteristics and Genetic Distances of ITS Sequences

The successfully sequenced ITS regions were approximately 644 bp in length. The average GC contents across all sequences was 58.35%, indicating relatively low overall variation and suggesting a structurally stable ITS region. Genetic distance analysis (Table S10) based on ITS sequences showed that all the genotypes exhibited genetic distances ranging from 0 to 0.0245, reflecting close phylogenetic relationships among all genotypes.

#### 2.1.2. Analysis of Polymorphic Sites

**Figure 1:**
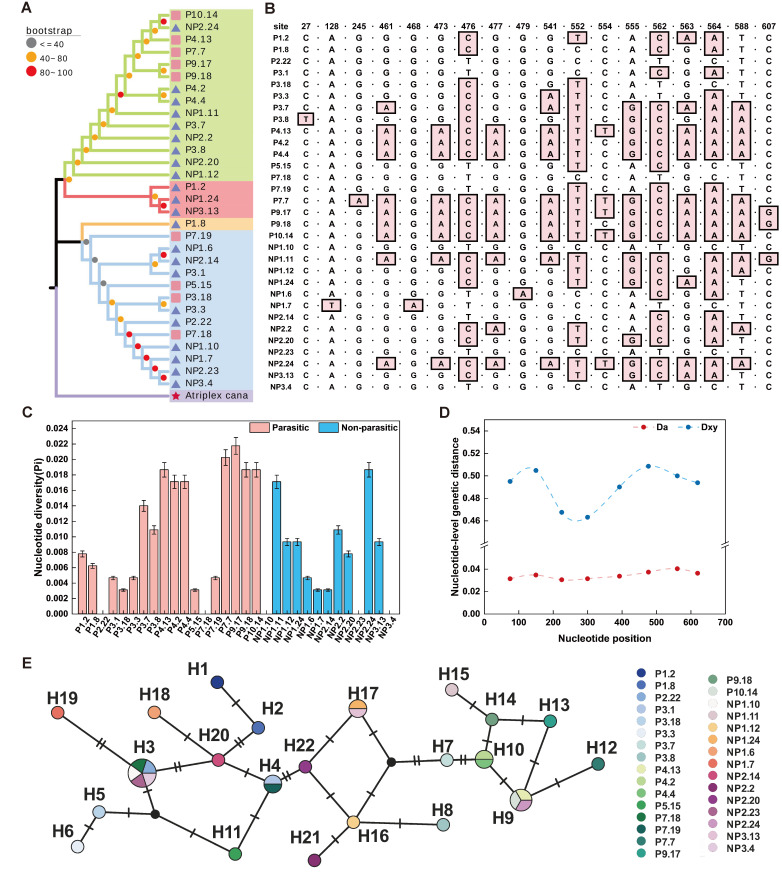
Analysis of genetic diversity among different genotypes of *A. canescens.* (**A**) A phylogenetic tree was constructed for 31 *A. canescens* genotypes. using the Neighbor-joining (NJ) method. (**B**) Characteristics of polymorphic sites in the ITS sequence. (**C**) Nucleotide diversity among 31 *A. canescens* genotypes. (**D**) Relationship between average nucleotide differences at sequence loci and net genetic distance. (**E**) Haplotype network analysis revealing 22 haplotypes, where different colors represent different genotypes.

Sequence alignment revealed two types of base deletions (1 bp and 2 bp) present in the ITS sequences across all genotypes. Analysis of polymorphic sites showed that genotypes P7.7 and P9.17 harbored the highest number of polymorphic sites (13), followed by P4.3, P9.18, P10.14, and NP2.24, each with 12 polymorphic sites. In contrast, genotypes P2.22, P7.18, NP1.10, NP2.23, and NP3.4 exhibited no detectable polymorphic sites, indicating relatively low levels of genetic variation. Overall, 29.03% of the genotypes (i.e., P4.13, P4.2, P4.4, P7.7, P9.17, P9.18, P10.14, NP1.11, and NP2.24) contained more than 10 polymorphic sites (Figure 1B), suggesting that these genotypes display comparatively high ITS sequence variability.

#### 2.1.3. Comparison of Genetic Diversity Among Genotypes

A significant positive correlation was observed between nucleotide diversity and the number of polymorphic sites among the genotypes. Nucleotide diversity increased synchronously with the accumulation of polymorphic sites (Figure 1C,D), indicating that polymorphic site variation is a major contributor to ITS sequence diversity.

Haplotype network analysis of ITS sequences from 31 *A. canescens* genotypes identified a total of 22 distinct haplotypes (Figure 1E). The haplotypes were relatively dispersed throughout the network and could be broadly classified into three clusters. Among them, H5 and H6 formed a distinct and isolated subclade corresponding to the closely related genotypes P3.18 and P3.3. Similarly, haplotypes H7, H9-H10, and H12-H15 were clearly separated from other haplotypes and corresponded to genotypes P3.7, P4.13, P10.14, NP2.24, P7.7, P9.17, P9.18, and NP1.11. These haplotypes exhibited close genetic relationships with one another but were more distantly related to other germplasm, a pattern consistent with the phylogenetic tree analysis.

The distribution of accessions among haplotypes varied considerably. Most haplotypes were unique to a single accession. Haplotypes H4, H10, and H17 were detected in two accessions each, while H9 was shared by three accessions (P4.13, P10.14, and NP2.24). H3 was the most widespread haplotype, occurring in P2.22, P7.18, NP1.10, NP2.23, and NP3.4. Overall, 22 haplotypes were identified among 31 *A. canescens* accessions, with the majority of haplotypes corresponding to single accessions. This pattern indicates a certain degree of genetic differentiation among the studied material at the ITS sequence level.

### 2.2. Phenotypic Diversity Among A. canescens Genotypes

#### 2.2.1. Plant Agronomic Traits

**Figure 2:**
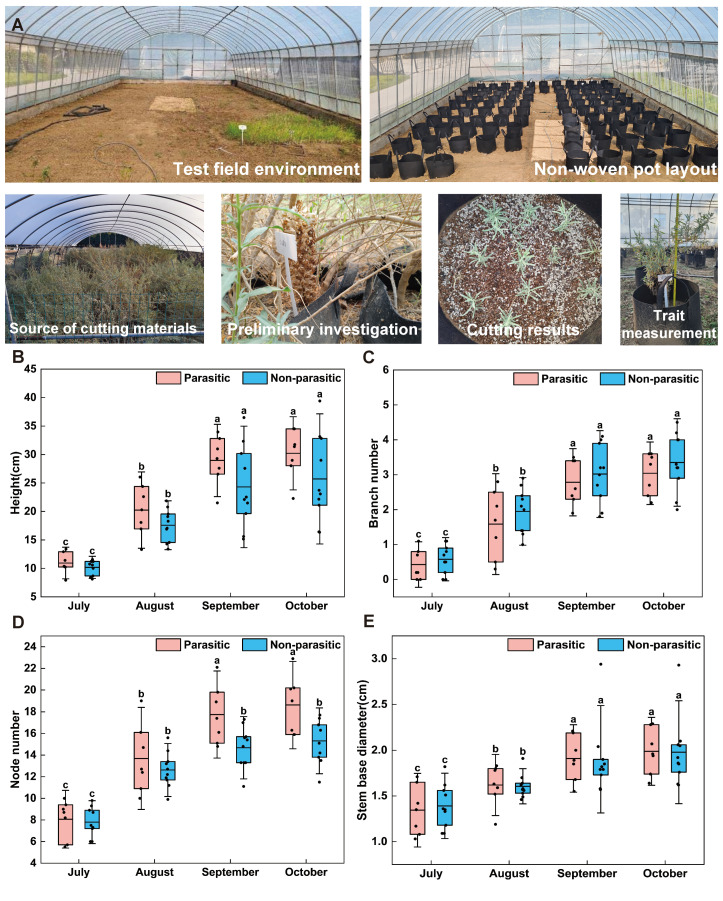
Greenhouse environment in the experimental field and growth of *A. canescens* from July to October. (**A**) Greenhouse construction, plant surveys, substrate preparation, and agronomic trait measurements. Agronomic trait indicators are shown as follows: (**B**) Plant height. (**C**) Branch Number. (**D**) Node Number. (**E**) Stem base diameter. Data are expressed as mean ± standard deviation (*n* = 7 for parasitic group, *n* = 10 for non-parasitic group). Red denotes the parasitized state, while blue denotes the non-parasitized state. Statistical analysis of differences among multiple groups was performed using one-way analysis of variance (ANOVA), followed by Duncan’s new multiple range test. Different lowercase letters indicate statistically significant differences (*p* < 0.05).

Growth dynamics, variation characteristics, and phenotypic diversity were evaluated for 17 *A. canescens* genotypes, with 10 plants analyzed per genotype. Four agronomic traits were assessed: plant height (H), branches number (BN), node number (NN), and stem base diameter (SBD) (Figure 2). The results showed that all traits increased highly significantly throughout the growing season from July to October (*p* < 0.001; Tables S1–S4). Overall, trait development followed a common pattern characterized by a “rapid growth phase–stable growth phase–growth stagnation phase.”

Among the evaluated traits, branch number exhibited the most pronounced growth, with a total growth rate of 589.85%. Growth peaked between August and September, during which the growth rate reached 280.77%, and the average branch number increased to 3.22 by October. Plant height showed a total growth rate of 168.37%, with the most rapid increase occurring from July to August (growth rate: 82.50%), ultimately reaching an average height of 27.57 cm by October. In contrast, the number of nodes and stem base diameter increased more moderately, with total growth rates of 118.56% and 46.44%, respectively. Both traits entered a growth stagnation phase between September and October, as indicated by growth rates below 12% (Table 1).

Notably, parasitic and non-parasitic *A. canescens* exhibited distinct growth patterns across different agronomic traits. Plant height, branch number and stem base diameter showed no significant differences among the three groups throughout the entire growth period. In contrast, the parasitic group maintained a significantly higher node number than the non-parasitic group from September to October (*p* < 0.05). By October, the mean node number of the parasitic group reached 18.61 ± 2.69, while that of the non-parasitic group was 15.31 ± 2.02.

Analysis of phenotypic variation revealed marked differences in trait dispersion within the population. Based on coefficients of variation (CV), traits ranked in decreasing order of variability as follows: BN (51.42%) > H (19.98%) > NN (17.94%) > SBD (14.27%) (Table 2). Notably, the CV of BN reached a maximum of 80.30% in September, indicating the greatest phenotypic differentiation during the rapid growth phase (August to September). H exhibited a relatively narrow CV range across months (14.69–24.33%), suggesting stable variation among genotypes and highlighting its suitability as a consistent selection index across different growth stages. Both SBD and NN showed low CV values with gradual temporal changes, reflecting strong genetic stability and phenotypic conservatism.

Further analysis of phenotypic diversity revealed that plant height exhibited the highest mean Shannon–Wiener diversity index (H′ = 1.5979), with a peak value of 1.7423 observed in August. This indicates substantial phenotypic differentiation among genotypes for plant height, underscoring its importance as a key trait for discriminating genetic materials.

**Table 1:** Comparison of Monthly Relative Growth Rates for Different Agronomic Traits.

Month	Plant Height Growth Rate (%)	Branching Growth Rate (%)	Node Number Growth Rate (%)	Stem Base Thickness Growth Rate (%)
July to August	77.5	256.3	66.6	20
August to September	45.5	70.2	28.5	22.2
September to October	4.4	8.2	2.9	4.5
Sum.	185	556.3	120.3	147.4

**Table 2:** Coefficient of Variation and Shannon-Wiener Diversity Index for Agronomic Traits of *A. canescens* in Different Months Across Genotypes.

Agronomic Traits	CV (%)					H′				
	July	August	September	October	Average	July	August	September	October	Average
H (cm)	14.69	19.64	24.33	21.47	19.98	1.48	1.74	1.69	1.48	1.60
BN (each)	23.02	39.33	80.30	63.04	51.42	1.30	1.32	1.35	1.38	1.42
NN (each)	16.78	18.65	18.64	18.69	17.94	1.51	1.40	1.32	1.45	1,34
SBD (mm)	10.36	14.10	14.84	17.78	14.27	1.47	1.33	1.14	1.15	1.27

Note: Average CV = (July CV + August CV + September CV + October CV)/4; Variability is ranked by the magnitude of the “average CV.” A higher average CV indicates greater dispersion of the trait among genotypes.

#### 2.2.2. Leaf Morphological Characteristics

**Figure 3:**
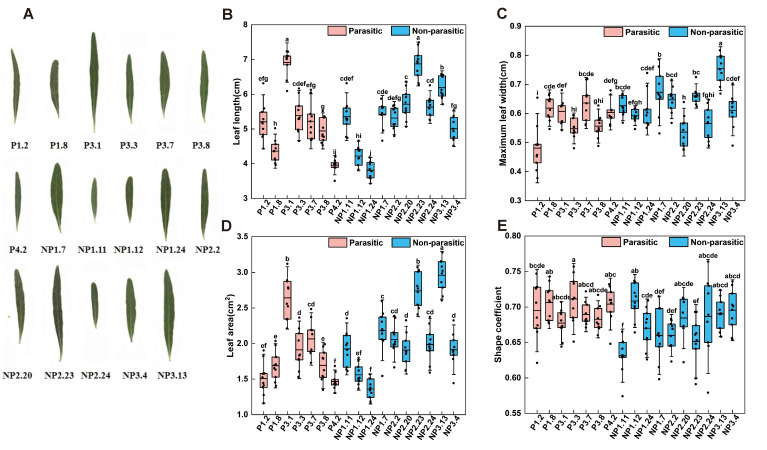
Morphological characteristics of leaves from 17 *A. canescens* genotypes. (**A**) Morphological characteristics of leaves from the 17 *A. canescens* genotypes; leaf traits are listed as follows: (**B**) leaf length; (**C**) maximum leaf width; (**D**) leaf square; (**E**) shape coefficient. Data are expressed as mean ± standard deviation (*n* = 7 for parasitic group, *n* = 10 for non-parasitic group, 10 leaves measured per genotype). Red denotes the parasitic state, while blue denotes the non-parasitic state. Statistical analysis of differences among multiple groups was performed using one-way analysis of variance (ANOVA), followed by Duncan’s new multiple range test. Different lowercase letters indicate statistically significant differences (*p* < 0.05).

Leaf morphological traits of 17 *A. canescens* genotypes were systematically scanned and analyzed. The results revealed significant differences among genotypes in leaf length, maximum width, leaf area, and shape coefficient (*p* < 0.01; Figure 3). However, there were no significant differences in leaf morphological traits between the parasitic and non-parasitic groups, indicating that the effect of *C. deserticola* parasitism on leaf length, maximum leaf width, leaf area, and shape coefficient was not significant. Most *A. canescens* leaves exhibited a narrow lanceolate morphology, with an average length-to-width ratio of approximately 8:1. The mean shape coefficient was 0.68 and showed the lowest coefficient of variation (CV = 4%), indicating relatively high stability of leaf shape among genotypes (Table 3).

In contrast, leaf area exhibited the highest coefficient of variation (CV = 19%), suggesting substantial differences in leaf size among genotypes and highlighting its potential as a key parameter for leaf classification. Based on leaf area and shape coefficient, leaves were classified into three categories (Table 4). Large leaves (area ≥ 2.50 cm^2^, shape coefficient ≥ 0.68) accounted for 17.6% of the samples; medium leaves (area 1.80–2.49 cm^2^, shape coefficient 0.66–0.68) accounted for 47.1%; and small leaves (area < 1.80 cm^2^, shape coefficient < 0.66) accounted for 35.3%. The distribution of leaf size categories was similar between parasitic and non-parasitic groups, further confirming that the observed variation in leaf traits may have arisen from the differences in genotypes rather than the effect of parasitism. Among the evaluated genotypes, NP3.13 exhibited the largest leaf area (2.96 cm^2^) and the greatest maximum width (0.75 cm). Genotype P3.1 showed the greatest leaf length (6.42 cm), whereas NP1.24 possessed the smallest leaf area (1.36 cm^2^) and the shortest leaf length (3.31 cm).

**Table 3:** Coefficient of Variation for Leaf Morphological Characteristics.

Indicator	AVG	SD	CV (%)
Leaf length (cm)	4.54	0.906	20
Maximum width (cm)	0.61	0.067	11
Leaf area (cm^2^)	1.97	0.374	19
Shape coefficient	0.68	0.027	4

**Table 4:** Classification and Proportion of Leaves in 17 *A. canescens* Genotypes.

Classification	Leaf Area Range	Shape Factor Range	Sample Number	Percentage
Large leaf	≥2.50	≥0.68	P3.1, NP2.23, NP3.13	17.6%
Medium leaf	1.80~2.49	0.66~0.68	P1.8, P3.3, P3.7, NP1.7, NP2.2, NP2.20, NP2.24, NP3.4	47.1%
Small leaf	<1.80	<0.66	P1.2, P3.8, P4.2, NP1.11, NP1.12, NP1.24	35.3%

### 2.3. Photosynthetic Physiological Diversity Among A. canescens Genotypes

**Figure 4:**
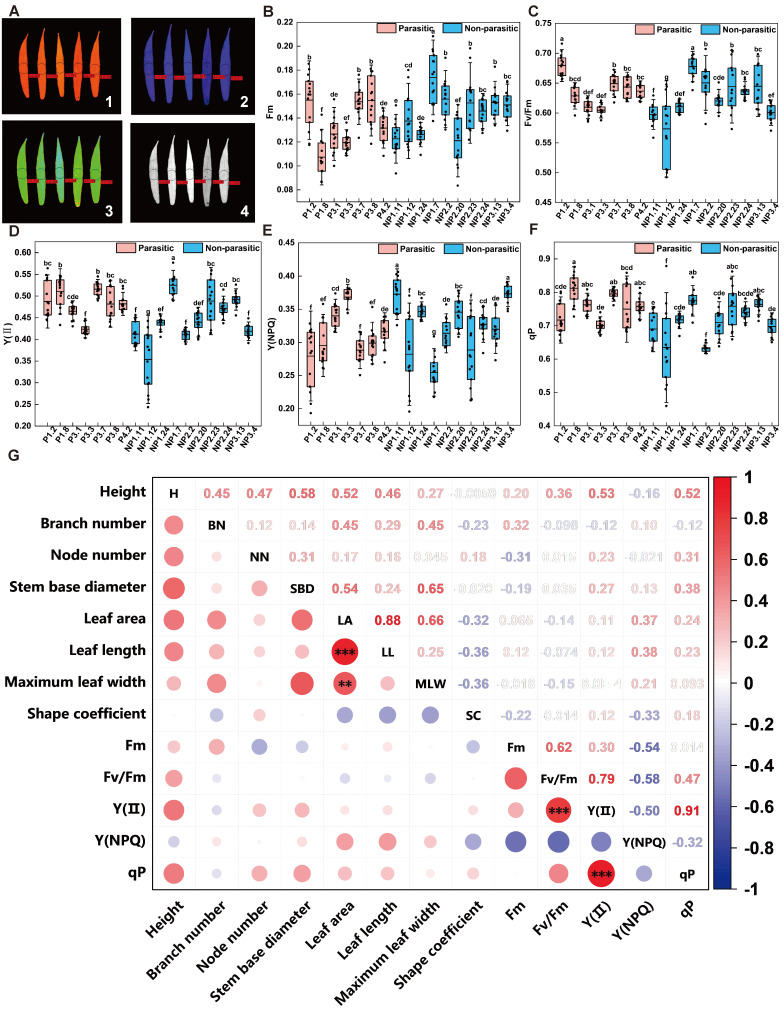
Analysis of chlorophyll fluorescence parameters in leaves from 17 *A. canescens* genotypes, along with correlation analysis of parasitic status, phenotypic traits, and physiological indicators. (**A**) Chlorophyll fluorescence imaging for leaves from the 17 *A. canescens* genotypes: (**1**) F_0_; (**2**) Fm; (**3**) Fv/Fm; (**4**) qP. The color scale in the figure intuitively reflects the variation in chlorophyll fluorescence parameters across samples. (**B**) Statistical parameters including: Fm, (**C**) Fv/Fm, (**D**) Y(II), (**E**) Y(NPQ), and (**F**) qP. Data are expressed as mean ± standard deviation (SD) (*n* = 7 for parasitic group, *n* = 10 for non-parasitic group, 15 leaves measured with 3 technical replicates per genotype). Red denotes the parasitic state, while blue denotes the non-parasitized state. Statistical differences among multiple groups were analyzed using one-way analysis of variance (ANOVA) followed by Duncan’s new multiple range test; different lowercase letters indicate statistically significant differences (*p* < 0.05). (**G**) Correlation analysis between phenotypic traits and physiological indicators: red denotes a positive correlation, while blue denotes a negative correlation. Significance was determined after Benjamini–Hochberg false discovery rate (FDR) correction for multiple testing (Significance levels: ** *p* < 0.01, *** *p* < 0.001).

The photosynthetic characteristics of 17 *A. canescens* genotypes were further analyzed (Figure 3A–F). Five key parameters reflecting photosystem II (PSII) function were evaluated: maximum fluorescence (Fm), maximum photochemical efficiency (Fv/Fm), actual photochemical efficiency [Y(II)], quantum yield of regulatory energy dissipation [Y(NPQ)], and photochemical quenching coefficient (qP). All five parameters exhibited significant inter-line differences (*p* < 0.05; Figure 4) and distinct variation patterns.

Fm, which reflects fluorescence intensity when the PSII reaction center is fully closed, ranged from 0.1073 to 0.1739, with an average value of 0.1401. Genotype NP1.7 exhibited the highest Fm value (0.1739), whereas P1.8 showed the lowest (0.1073). Fv/Fm, representing the potential photochemical efficiency of PSII, ranged from 0.5632 to 0.6790, with an average of 0.6318. The Fv/Fm value of genotype NP1.12 was significantly lower than that of other genotypes, decreasing by approximately 17.3% relative to the maximum value, suggesting possible stress-induced impairment of its PSII reaction centers.

Y(II), which indicates actual light energy conversion efficiency, ranged from 0.3537 to 0.5249, with an average of 0.4623. Genotypes NP1.7, P3.7, and P1.8 exhibited relatively high photosynthetic potential. Y(NPQ), reflecting photoprotective capacity, ranged from 0.2547 to 0.3731, with an average of 0.3152. Genotypes NP3.4 and NP1.11 showed the strongest photoprotection ability. The qP value, which characterizes electron transport efficiency, ranged from 0.6322 to 0.8121, with an average of 0.7345. Higher qP values were observed in P1.8, P3.7, and NP1.7, indicating stronger electron transport capacity in these genotypes.

Further comparisons between the parasitic group (P lines) and the non-parasitic group (NP lines) revealed that P lines generally outperformed NP lines in several photosynthetic parameters. The P series exhibited higher mean values of Fv/Fm (0.6359), Y(II) (0.4809), and qP (0.7523) than the NP series (0.6251, 0.4501, and 0.7227, respectively). Moreover, 71.4% of P-line individuals displayed Fv/Fm values above the overall mean, significantly exceeding the proportion observed in NP lines (30.0%). P lines also demonstrated a clear advantage in Y(II), indicating that Cistanche-parasitized individuals generally possess enhanced photosynthetic efficiency.

### 2.4. Correlation Analysis Among Traits in Different A. canescens Genotypes

To investigate associations among agronomic traits, leaf morphological characteristics, and chlorophyll fluorescence parameters in *A. canescens*, Pearson correlation analyses were performed across 17 genotypes (Figure 4G).

Significant intrinsic correlations were observed among leaf morphological traits. Leaf area exhibited highly significant positive correlations with both leaf length (*r* = 0.88, *p* < 0.001) and leaf width (*r* = 0.66, *p* < 0.01), indicating that these two parameters are the primary determinants of leaf area. In contrast, the correlation between leaf length and leaf width was weak and non-significant. Analysis of chlorophyll fluorescence parameters showed that Y(II) was highly positively correlated with Fv/Fm *(r* = 0.79, *p* < 0.001) and qP (*r* = 0.91, *p* < 0.001), while exhibiting a negative correlation with Y(NPQ) (r = -0.50). This pattern reflects a trade-off between photosynthetic energy utilization and regulatory heat dissipation. These results suggest that plant growth vigor is closely linked to photosynthetic functional activity.

### 2.5. TOPSIS Comprehensive Evaluation

**Figure 5:**
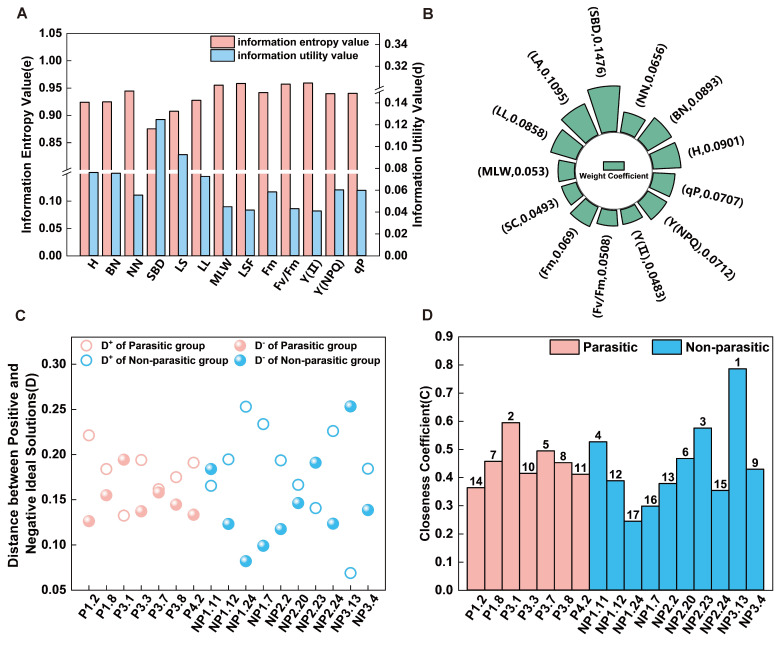
TOPSIS Comprehensive Evaluation of 17 *A. canescens* genotypes. (**A**) Information entropy and information utility values of parasitic status, phenotypic traits, and physiological indicators; (**B**) weight coefficients of parasitic status, phenotypic traits, and physiological indicators; (**C**) positive and negative ideal solution distances for 17 *A. canescens* genotypes: Positive ideal solution distance (*D^+^*); Negative ideal solution distance (*D^-^*); (**D**) Ranking of relative proximity to the ideal solution for the 17 *A. canescens* genotypes, where red denotes the parasitic state and blue denotes the non-parasitic state.

To identify superior host genotypes of *A. canescens*, the present study integrated the results of correlation analyses between phenotypic traits and parasitic performance. The entropy weight method was applied to determine the weights of individual indicators (Table S7), and the Euclidean distances to the positive (*D^+^*) and negative (*D^−^*) ideal solutions were calculated for each genotype (Table S8). Based on these values, a comprehensive evaluation index (*Ci*) was derived. The results showed that *Ci* values among the tested genotypes ranged from 0.1556 to 0.7577. When ranked in descending order of *Ci* values (Figure 5), the top three genotypes—NP3.13, P3.1, and NP2.23—demonstrated superior overall performance, characterized by strong growth potential and high host suitability. These genotypes therefore represent promising candidate host genotypes for *C. deserticola* production, with potential for prioritized cultivation and preliminary evaluation. To validate the robustness of our findings, we used the CRITIC method [24] as an alternative weighting strategy (Figure S1), which had a Spearman rank correlation coefficient of 0.928 (*p* < 0.001) with the TOPSIS rankings, indicating a high level of consistency (Table S12).

## 3. Discussion

### 3.1. Intraspecific Diversity and Ecological Adaptation of A. canescens

*A. canescens* exhibits noticeably intraspecific diversity at both phenotypic and genetic levels, which is closely associated with its ecological adaptation strategies [28].

At the phenotypic level, marked variations were observed in agronomic traits and leaf morphological characteristics among genotypes, and these differences show ecological adaptive value. Narrow lanceolate leaves with low morphological variability represent a conservative and stable strategy for drought adaptation, as such leaf forms reduce transpiration and water loss in arid environments [29]. In contrast, the relatively high variations in leaf area reflect functional differentiation of different genotypes in terms of resource utilization efficiency [30]. Due to the limitation of sample size, the quantitative analysis of phenotypic diversity in this section is only regarded as a preliminary observation.

At the genetic level, haplotype network analysis initially linked phenotypic variations with genetic backgrounds. The genotypes of the dominant haplotype H3 exhibited better genetic stability and phenotypic coordination, among which genotype NP2.23 was identified as an elite representative, suggesting that haplotype H3 may be related to enhanced environmental adaptability and provides insight into the genetic basis underlying phenotypic advantages. In addition, the presence of several isolated minor groups likely resulted from the geographic isolation or local habitat selection after introduction [31], which can serve as genetic resources for mining special adaptive traits. Compared with previous studies that mainly focused on morphological variations [5,6], this study initially revealed the phenotypic diversity and ITS-based genetic differentiation of *A. canescens*, and preliminarily explored the correlation between phenotypic traits and genetic background.

### 3.2. Correlation Mechanisms Between Key Traits and Host Suitability for C. deserticola

The host suitability of *A. canescens* for *C. deserticola* is jointly regulated by agronomic traits, leaf morphology, and photosynthetic physiology. These traits interact synergistically to determine the capacity of the host to supply nutrients to parasitic plants.

In terms of leaf morphology and function, leaves are the key organs mediating plant adaptation and photosynthetic performance [32,33,34,35]. The leaf morphology shows the characteristics of “size differentiation with shape conservation”, which reflects adaptive responses to habitat conditions [36,37]. Variation in leaf area indicates differentiation among genotypes in light resource acquisition strategies [38,39], whereas the stable narrow leaf shape may enhance drought tolerance by reducing water transpiration. Superior host genotypes typically exhibit an optimized photosynthetic area, providing structural support for nutrient supply to the parasitic plant. Chlorophyll fluorescence parameters show that the photosynthetic system activity of the parasitic group of *A. canescens* is better, which is adapted to parasitic stress through optimized light energy allocation strategies. Different genotypes display distinct physiological strategies that are closely associated with their genetic backgrounds, and some of them can balance the requirements between self-growth and nutrient export to the parasite [40].

Overall, leaf area establishes the structural foundation for photosynthetic production, while an efficient photosynthetic system ensures the accumulation of assimilates. These traits support effective nutrient supply to *C. deserticola*, providing a theoretical basis for trait-oriented selection of *A. canescens* host materials.

### 3.3. Innovative Value and Application Prospects of the Comprehensive Evaluation System

Traditional host screening often relies on single traits, such as plant height or parasitism rate, which are subjective and insufficient to capture the comprehensive performance of host plants. In contrast, this study integrates genetic diversity, phenotypic traits, and photosynthetic physiology, applying entropy weight method to objectively assign weights and quantitatively ranking the genotypes in combination with the TOPSIS model for quantitative genotypes ranking. This approach largely reduced the subjectivity of weight allocation during multi-index evaluation.

Analysis of indicator contributions revealed that plant height, stem base diameter, and Y(II) are the primary determinants influencing comprehensive evaluation outcomes, which identify key target traits for host selection and provide clear guidance for following breeding programs. The superior genotypes identified—NP3.13, P3.1, and NP2.23—not only exhibit strong growth vigor and high photosynthetic efficiency but also possess unique haplotype characteristics (H3 and H9). NP (non-parasitized) status in this study refers to the phenotypic condition in which *A. canescens* plants were not successfully parasitized by *C. deserticola* under initial field cultivation conditions. This status is mainly caused by non-genetic environmental factors, including field climate, inoculation timing, randomness of seed distribution, and haustorium formation of the parasite, rather than genetic incompatibility or an intrinsic lack of parasitic capacity in *A. canescens*. Therefore, the NP classification did not affect the interpretation of correlation analyses, which focused on intrinsic host traits and were adjusted by FDR correction. The designation of superior hosts was based on comprehensive evaluation of agronomic, physiological, and photosynthetic traits using the TOPSIS method, rather than parasitism status alone. NP genotypes identified as superior hosts exhibited excellent growth and physiological performance, which provides the essential material and energy foundation for successful parasitism. Thus, this transient non-parasitic phenotype did not compromise the reliability of host evaluation, and the classification of superior hosts was fully justified biologically and experimentally.

Moreover, the comprehensive evaluation system established in this study offers methodological insights applicable to host screening in other parasitic plant, such as *Santalum album*, *Orobanche coerulescens*. This framework can be flexibly adapted to diverse parasitic interactions, addressing the industry pain point of the lack of standardized methods for host selection in parasitic plant cultivation.

### 3.4. Limitations and Future Prospects

Although this study has achieved certain progress in germplasm evaluation and identifying superior host genotypes of *A. canescens*, several limitations should be acknowledged.

Firstly, the sample size was relatively limited, with only 17 genotypes included in the core evaluation, which may not fully capture the breadth of genetic and phenotypic variation in *A. canescens*. Notably, these 17 genotypes were randomly selected from the surviving individuals after multiple rounds of cutting propagation, and such a survival-based selection process could be easily affected by environmental factors, which may further influence the representativeness of the experimental materials. And the correlation analysis was conducted with a relatively small sample size, which may lead to unstable or less reliable correlation coefficients. Therefore, the results should be interpreted with caution, and further studies with larger sample sizes are needed to validate these relationships.

Secondly, this study lacks direct quantitative data on parasitism rate, yield performance, and key pharmacologically active compounds (e.g., phenethyl glycosides and polysaccharides) in *C. deserticola* after inoculation. The classification of genotypes as having “high parasitic potential” was therefore only indirectly inferred from host growth and photosynthetic traits, without validation from practical cultivation experiments, and represents a key focus for improvement in future studies. Thirdly, the regulatory effects of environmental factors, including soil salinity, water availability, and microbial community composition, were not systematically examined, and these factors may significantly influence the field performance of different genotypes [41]. Finally, our genetic diversity analysis was solely based on ITS sequences, which have relatively low resolution for intraspecific investigations and can only reflect genetic differentiation within the ribosomal DNA region. We were unable to dissect the comprehensive genetic diversity, fine-scale population structure, or the genetic mechanisms underlying the elite traits of candidate host genotypes.

To address these limitations, future research should focus on several key directions. Firstly, germplasm collection should be expanded to include *A. canescens* genotypes from different introduction sources and habitat types. The integration of high-throughput molecular markers, such as simple sequence repeats (SSRs) and single-nucleotide polymorphisms (SNPs), would facilitate the construction of a more comprehensive genetic diversity map [42,43]. Secondly, carry out potted experiments and large-scale field trials involving inoculation with *C. deserticola*, systematically quantify parasitism rates, host biomass accumulation, and medicinal component contents (e.g., phenethyl glycosides and polysaccharides), thereby directly validating parasitic benefits. Thirdly, integrate transcriptomics, metabolomics, and quantitative trait locus (QTL) mapping to identify key regulatory genes (e.g., those involved in photosynthetic regulation and root development) and molecular markers associated with host suitability, enabling molecular-assisted selection and breeding [44]. Fourthly, explore the interactive effects of environmental factors and host traits, and establish region-specific cultivation strategies. Finally, long-term field trials are necessary to assess the genetic stability, ecological adaptability, and sustained parasitic performance of superior genotypes, providing essential data support for industrial-scale application.

## 4. Materials and Methods

### 4.1. Experimental Materials and Cultivation Background

The *A. canescens* samples originated from over 300 one-year-old seedlings purchased by the research team from Huarui Landscape Gardening Co., Ltd. in Jingbian County, Shaanxi Province in 2021. After the planting was stable, seeds of *C. deserticola* were quantitatively inoculated manually. Initial genotype selection was conducted based on plant survival rates and parasitism status. Selected plants were subsequently introduced to the Beijing experimental field of the Institute of Medicinal Plant Development, Chinese Academy of Medical Sciences, for continued propagation in 2022. Based on previous investigations of growth characteristics and parasitism status, 17 distinct samples exhibiting characteristic variations were selected from the existing 31 genotypes and numbered according to their original row-column sequence at planting (Table 5).

Substrate preparation and cuttings propagation were conducted in the experimental field on 14–15 May 2025. The cultivation substrate composed of nutrient soil and vermiculite mixed uniformly at a 1:2 volume ratio was loaded into felt pots (40 cm height, 50 cm diameter). These pots were arranged in the cultivation greenhouse (greenhouse specifications: length 7.4 m, width 14.7 m) with 30 cm spacing between rows and substrates respectively. Each pot was planted 16 cuttings, with 4 replicate pots for each genotype. Semi-lignified vigorous branches were selected as scions and segments with 2~4 buds and 1~2 leaves at the apex were cut as cuttings of uniformly 15 cm in length. Before planting, cutting bases were soaked in 1% ABT rooting powder solution and 1% carbendazim solution for 1 h. Cuttings were inserted to a depth of one-third of their length, with 1–2 buds exposed above ground. After insertion, the substrate was compacted, cuttings were numbered, and field management was implemented afterward.

The growth status survey of the cuttings revealed all plants were healthy, vigorous, and maintained stable growth on 14 June 2025. From July to late October 2025, agronomic trait measurements and leaf sampling were conducted. Uniform field management was implemented throughout the trial period to maintain consistent cultivation conditions. All physiological and photosynthetic traits were measured under non-parasitic conditions.

### 4.2. Measurement Indicators and Methods

#### 4.2.1. ITS Molecular Marker

Healthy, uniformly sized and representative mature leaves were sampled from 31 extant *A. canescens* plants of distinct genotypes (genotype numbers listed in Table 6) for genomic DNA extraction. After sterilization with 75% ethanol, fresh leaves were placed in 2 mL centrifuge tubes and homogenized using a cryogenic grinder For Total DNA extraction and sequencing.

##### The Method of DNA Extraction

Genomic DNA was extracted from fresh leaf tissues of *A. canescens* using the TIANGEN plant genomic DNA extraction kit (TIANGEN Biotech Co., Ltd., Beijing, China), following the manufacturer’s instructions. DNA concentration and purity were examined using a NanoDrop ND-1000 spectrophotometer (Thermo Fisher Scientific, Wilmington, DE, USA). The ITS region was amplified and sequenced by Sangon Biotech Co., Ltd. (Shanghai, China) or the Major Platform Center, Institute of Crop Sciences, Chinese Academy of Agricultural Sciences (Beijing, China). PCR amplification was performed with three technical replicates per genotype to ensure reliability and reproducibility. PCR products were detected by 2% agarose gel electrophoresis, and fragments of 650–700 bp were recovered for Sanger sequencing.

The ITS region was amplified with the PCR primers ITS1 (5′-TCCGTAGGTGAACCTGCGG-3′) and ITS4 (5′-TCCTCCGCTTATTGATATGC-3′).

PCR amplification program: 94 °C pre-denaturation for 5 min; 35 cycles of 94 °C denaturation for 50 s, 55 °C annealing for 30 s, 72 °C extension for 90 s; final extension at 72 °C for 10 min.

#### 4.2.2. Agronomic Traits of Different Plant Genotypes

From July to October 2025, agronomic traits of *A. canescens* were investigated every 30 days (n = 7 for parasitic group, n = 10 for non-parasitic group). Ten healthy plants were randomly selected from each genotype to measure plant height (H), branch number (BN), node number (NN), and stem base diameter (SBD):

Plant height: Vertical distance measured with a tape measure from the ground level rhizome to the highest point of the plant in its natural state (cm);

Branch Number: Count the number of primary branches emerging from the plant base (number);

Node Number: Count the number of nodes between the first node at the base and the terminal node on the main stem (number);

Stem Base Diameter: Measure the diameter (mm) at the base of the main stem, immediately above ground level, using an electronic digital caliper (0.01 mm precision).

#### 4.2.3. Leaf Morphological Characteristics

On 9 October 2025, ten mature leaves that were healthy, uniform in size, and representative per plant genotype were selected and scanned using the broadleaf analysis software WinFOLIA to measure four parameters: leaf length (LL), maximum leaf width (MLW), leaf area (LA), and shape coefficient (SC) (n = 7 for parasitic group, n = 10 for non-parasitic group, 10 leaves measured per genotype).

#### 4.2.4. Chlorophyll Fluorescence Parameters

At 7:00 AM, on 15 September 2025, mature leaves at the same leaf position were selected from each genotype with consistent light conditions, exhibiting healthy and similar vigor (n = 7 for parasitic group, n = 10 for non-parasitic group, 15 leaves measured with 3 technical replicates per genotype). Fifteen leaves per genotype were tested with three replicates. Chlorophyll fluorescence parameters were measured using the IMAGING-PAM chlorophyll fluorescence imaging system (Heinz Walz GmbH, Effeltrich, Germany). Before measurement, leaf surfaces were cleaned with lens paper and dark-adapted for 30 min. Leaves were then placed on the imaging system’s stage to acquire fluorescence images, with relevant parameters read from designated regions.

During the measurement process, the initial fluorescence value (F_0_) and maximum fluorescence value (Fm) were first determined before activating the excitation light. Subsequently, a series of saturating flash pulses (6000 μmol·m^−2^·s^−1^, pulse duration 2 s) was applied under natural light, with measurements taken every 20 s until the pulses ceased. The mean value of the last 6 saturated flash pulse measurements was used to calculate the maximum fluorescence (Fm), maximum photochemical efficiency (Fv/Fm), actual photochemical efficiency [Y(II)] and photochemical quenching coefficient (qP) parameters.

### 4.3. Data Processing and Analysis Methods

#### 4.3.1. ITS Sequence Analysis

In this study, the ITS sequences were assembled and corrected using CodonCode Aligner 12.0.4 (Codon Code Corporation, Centerville, MA, USA), with low-quality sequences at both ends removed. The ITS sequences were aligned using MEGA12 [45] to calculate sequence lengths and nucleotide compositions, and compute genetic distances among different genotypes. A phylogenetic tree was constructed using the Neighbor-joining method, and the tree was visualized using the EvolView v3 [46]. DNAsp v5 [47] was used to calculate polymorphic sites, haplotype diversity, and nucleotide diversity. Haplotype network analysis was conducted using Arlequin ver 3.5.2.2 [48] and PopART V1.7 [49]. OriginPro 2025b (OriginLab Corporation, Northampton, MA, USA) was utilized to draw relevant statistical charts.

#### 4.3.2. Data Processing and Statistical Analysis

The agronomic traits, leaf morphological characteristics, and chlorophyll fluorescence parameter data were analyzed using Microsoft Excel 2019 (Microsoft Corporation, Redmond, WA, USA) and IBM SPSS Statistics 27.0.1 (IBM Corp., Armonk, NY, USA) to calculate the mean (AVG), standard deviation (SD), and coefficient of variation (CV, %). Box plots were drawn using OriginPro 2025b. Differences in traits among different genotypes were tested using one-way analysis of variance (ANOVA), followed by Duncan’s multiple range test for mean comparisons at the *p* ≤ 0.05 level to identify statistically significant differences between genotypes.

To quantify phenotypic diversity, the Shannon-Wiener diversity index (H′) was used for evaluation. Higher H′ values indicate greater phenotypic diversity among genotypes for the trait [25], calculated as follows:

$$H^{\prime }=-\sum_{i=1}^{S}\left(P_{i}\ln P_{i}\right)$$

H′: Shannon-Wiener diversity index;

Pi: Frequency of the i^(th) phenotypic group (number of genotypes in group/total number of genotypes);

S: Number of phenotypic groups, determined based on trait distribution characteristics;

In: Natural logarithm.

Additionally, Pearson correlation coefficients were used to analyze relationships between indicators. Correlation analysis was performed using the Correlation Plot plugin in OriginPro 2025b. Microsoft Excel 2019 was used to organize phenotypic and photosynthetic physiological data, calculate Pearson correlation coefficients, and perform the Benjamini–Hochberg false discovery rate (FDR) correction for multiple testing.

#### 4.3.3. TOPSIS Model for Comprehensive Evaluation

This study used the Entropy Weighted TOPSIS model for multi-indicator comprehensive evaluation, aiming to screen *A. canescens* genotypes with high parasitic potential and vigorous growth. Firstly, traits related to host suitability were selected. Entropy weighting was applied to calculate and normalize the weights of each indicator. Next, the maximum and minimum values for each evaluation indicator were determined. Quantitative assessments of genotypes with varying traits and parasitic statuses were conducted by calculating the Euclidean distance *D_i_^+^* from sample i to the ideal state (Formula (1)) and the Euclidean distance *Di^-^* from sample i to the worst state (Formula (2)). This enabled the computation of the comprehensive score *Ci* (Formula (3)) for ranking the overall performance of different genotypes.

(1) $$D_{i}^{+}=\sqrt{\sum_{j=1}^{m}(Z_{\max\;j}-Z_{\mathrm{ij}}{)}^{2}}$$

(2) $$D_{i}^{-}=\sqrt{\sum_{j=1}^{m}(Z_{\mathrm{ij}}-Z_{\min\;j}{)}^{2}}$$

(3) $$C_{i}=\frac{D_{i}^{-}}{D_{i}^{+}+D_{i}^{-}}$$

Formulas (1)–(3): Z represents the ideal solution, *Di^+^* and *Di^−^* represent distances, and *Ci* represents the comprehensive score [20].

## 5. Conclusions

In summary, this study provides a dual contribution: first, it establishes a robust, multi trait evaluation framework that integrates genetic, phenotypic, and photosynthetic parameters for systematically screening host germplasm; second, applying this framework, we identified three genotypes (NP3.13, P3.1, and NP2.23) with superior growth vigor and physiological performance as promising candidate hosts for *C. deserticola* cultivation, although their practical value requires further verification by direct parasitism and yield measurements. The methodology developed here not only supports the targeted selection of high-potential hosts for this parasitic species but can also be adapted for host screening in other parasitic plant systems. Looking forward, the large-scale deployment of these selected genotypes, coupled with further validation in field trials, holds promise for enhancing the yield and quality of *C. deserticola*, while contributing to the ecological and economic sustainability of desert region agriculture. This study not only provides core germplasm materials and methodological support for *C. deserticola* host selection but also offers significant references for studying parasitic plant–host interaction mechanisms and evaluating introduced plant germplasm.

## Figures and Tables

**Table 5:** Identification Numbers and Parasite Status of 17 Different Genotypes of *A. canescens*.

NO.	Location	Status	NO.	Location	Status	NO.	Location	Status
1	1.2	P	7	4.2	P	13	2.20	NP
2	1.8	P	8	1.7	NP	14	2.23	NP
3	3.1	P	9	1.11	NP	15	2.24	NP
4	3.3	P	10	1.12	NP	16	3.4	NP
5	3.7	P	11	1.24	NP	17	3.13	NP
6	3.8	P	12	2.2	NP			

**Note**: **Location**: The *A. canescens* samples original row-column sequence at planting; Status: **P** denotes plants parasitized by *C. deserticola* under cultivation conditions; **NP** denotes plants not yet parasitized under cultivation conditions; The **P/NP** labels represent fixed classification based on prior infection history, rather than real-time active parasitism during the greenhouse assessment.

**Table 6:** Identification Numbers and Parasite Status of 31 Different Genotypes of *A. canescens*.

NO.	Location	Status	NO.	Location	Status	NO.	Location	Status
1	1.2	P	12	7.18	P	23	1.12	NP
2	1.8	P	13	7.19	P	24	1.24	NP
3	2.22	P	14	7.7	P	25	2.2	NP
4	3.1	P	15	10.14	P	26	2.14	NP
5	3.3	P	16	4.13	P	27	2.20	NP
6	3.7	P	17	3.18	P	28	2.23	NP
7	3.8	P	18	5.15	P	29	2.24	NP
8	4.2	P	19	1.6	NP	30	3.4	NP
9	4.4	P	20	1.7	NP	31	3.13	NP
10	9.17	P	21	1.10	NP			
11	9.18	P	22	1.11	NP			

**Note**: **Location**: The *A. canescens* samples original row-column sequence at planting; Status: **P** denotes plants parasitized by *C. deserticola* under cultivation conditions; **NP** denotes plants not yet parasitized under cultivation conditions; The **P/NP** labels represent fixed classification based on prior infection history, rather than real-time active parasitism during the greenhouse assessment.

## Data Availability

The original contributions presented in this study are included in the article/supplementary material. Further inquiries can be directed to the corresponding authors.

## Supplementary Materials

The following supporting information can be downloaded at: https://www.mdpi.com/article/10.3390/plants15060881/s1, Table S1: Changes in Plant Height by Month; Table S2: Changes in Plant Branch Number by Month; Table S3: Changes in Plant Node Number by Month; Table S4: Changes in Stem Base Diameter of Plants Across Different Months; Table S5: Comparison of Leaf Characteristics Among *Atriplex canescens* genotypes; Table S6: Comparison of Chlorophyll Fluorescence Parameters Among *Atriplex canescens* genotypes; Table S7: Weighting of Factors Affecting *Atriplex canescens* Growth Calculated Using Entropy Weighting Method; Table S8: TOPSIS-Based Comprehensive Evaluation Ranking of *Atriplex canescens* genotypes; Table S9: Base Sequence Characteristics of *Atriplex canescens* genotypes; Table S10: Genetic Distances Among Different Clones of *Atriplex canescens* genotypes; Table S11: Analysis of Genetic Diversity Among Different genotypes. Table S12: The rankings of 17 *Atriplex canescens* genotypes under both methods; Figure S1: CRITIC method Evaluation of 17 *Atriplex canescens* genotypes.
