# Supplementary material for: Assessment of Intraspecific Diversity and Screening of Elite Genotypes of Atriplex canescens as a Host Plant for Cistanche deserticola
Source: Plants (Basel). 2026 Mar 12;15(6):881. doi: 10.3390/plants15060881 (PMC13030436; doi:10.3390/plants15060881)
Supplement: Supplementary file 1 [file plants-15-00881-s001.zip › plants-4171179-supplementary.pdf]

Supplementary Table S1. Changes in Plant Height by Month

NO.	July	August	September	October
P1.2	10.38±1.22cdef	16.94±7.24efgh	26.55±3.01f	28.03±2.71d
P1.8	11.41±2.43abc	20.28±4.98bcde	29.30±3.88def	31.39±2.35c
P3.1	10.30±1.75cdef	24.39±4.83ab	32.81±4.38bc	34.53±2.37b
P3.3	7.93±2.45g	22.60±2.76abc	27.59±2.47ef	28.98±2.96d
P3.7	12.93±2.53ab	13.32±3.18h	30.99±1.75cd	31.75±1.58c
P3.8	13.39±2.85a	26.05±3.61a	33.96±2.38ab	34.51±1.99b
P4.2	10.24±2.56cdef	18.07±4.78defg	21.51±3.23g	22.28±2.17ef
NP1.11	8.44±1.35efg	20.79±4.45bcde	32.33±2.41bc	33.15±2.13bc
NP1.12	10.76±1.13bcd	19.56±2.41cdef	21.49±0.96g	22.16±1.41ef
NP1.24	8.68±2.86defg	14.56±2.86gh	15.16±3.24h	16.35±2.38g
NP1.7	11.22±2.70abc	16.90±3.03efgh	22.10±2.63g	23.08±2.06ef
NP2.2	11.23±2.67abc	19.10±3.68cdef	19.63±1.30g	21.11±1.29f
NP2.20	8.12±2.30fg	21.87±5.14bcd	30.18±2.84cde	32.83±2.21bc
NP2.23	11.57±2.67abc	17.06±4.40efgh	27.57±3.29ef	28.99±2.45d
NP2.24	10.59±1.68cde	13.39±3.63h	15.61±2.48h	16.41±2.10g
NP3.4	11.20±1.52abc	18.28±4.33cdefg	22.53±2.78g	23.72±1.63e
NP3.13	10.02±2.29cdefg	14.30±5.60fgh	36.49±4.63a	39.40±3.98a
Average	10.49	18.75	26.22	27.59
Equality of Variances	4.695**	7.085**	46.537**	83.732**

Note: N=10, Different lowercase letters indicate significant differences (p<0.05), **indicates extremely significant differences (p<0.01)

Supplementary Table S2. Changes in Plant Branch Number by Month

NO.	July	August	September	October
P1.2	0.00±0.00c	0.30±0.48g	2.30±1.42de	2.40±0.84def
P1.8	0.20±1.10ab	1.20±1.03defg	1.90±0.74e	2.20±0.79ef
P3.1	0.70±0.48abc	2.10±1.10abcd	2.60±0.97cde	3.30±1.06bcde
P3.3	0.00±0.00c	2.80±1.23a	3.40±1.26abcd	3.60±1.71abcd
P3.7	0.20±0.63bc	0.50±0.85fg	3.40±1.35abcd	3.50±1.08abcd
P3.8	1.10±1.20a	2.50±0.97ab	3.50±1.08abc	3.60±1.71abcd
P4.2	0.80±0.79ab	1.70±0.48bcde	2.40±0.52cde	2.70±0.82def
NP1.11	0.70±0.67abc	2.30±1.25abc	3.90±1.37ab	4.50±1.18a
NP1.12	0.00±0.00c	2.90±0.74a	3.20±0.63abcd	3.30±0.95bcde
NP1.24	0.80±0.42ab	2.00±0.94abcd	3.00±0.94bcd	3.20±1.23bcde
NP1.7	1.10±0.99a	2.10±1.10abcd	3.20±1.03abcd	3.20±1.03bcde
NP2.2	0.50±0.85abc	1.40±0.52cdef	1.90±1.10e	2.20±1.03ef
NP2.20	1.10±0.74a	2.70±0.67a	2.70±0.95cde	2.90±1.10cdef
NP2.23	0.00±0.00c	2.40±1.07ab	4.00±1.05ab	4.00±1.33abc
NP2.24	0.50±0.71abc	1.00±1.05efg	1.80±0.92e	2.00±0.67f
NP3.4	0.20±0.42bc	1.30±1.42def	4.10±1.37a	4.20±1.23ab
NP3.13	0.90±0.57ab	1.40±0.70cdef	2.40±0.70cde	4.00±1.25abc
Average	0.56	1.8	2.92	3.22

Equality of Variances	3.794**	6.706**	4.969**	4.142**

Note: N=10, Different lowercase letters indicate significant differences (*p*<0.05), **indicates extremely significant differences (*p*<0.01)

Supplementary Table S3. Changes in Plant Node Number by Month

NO.	July	August	September	October
P1.2	8.70±1.42abc	19.00±2.87a	19.80±2.49ab	20.10±2.92ab
P1.8	5.50±2.07d	12.70±3.71def	17.40±3.24bcd	20.20±3.08ab
P3.1	10.00±1.56a	12.40±2.17defg	22.10±3.07a	22.90±2.81a
P3.3	5.70±2.41d	14.70±1.77bcd	16.10±3.21cde	16.30±3.06cdef
P3.7	9.00±1.70abc	10.90±3.87fg	15.10±3.41de	15.90±4.41cdef
P3.8	9.40±2.63ab	10.00±3.20g	14.80±2.04de	15.90±2.38cdef
P4.2	8.20±3.29abc	16.10±1.66b	18.90±4.82bc	19.00±4.50bc
NP1.11	6.00±1.41d	12.20±2.25defg	17.00±1.83bcd	17.40±3.50bcd
NP1.12	8.90±1.45abc	13.40±1.71cdef	15.40±0.97de	16.80±1.62cde
NP1.24	7.30±2.91bcd	11.20±1.75fg	14.80±2.35de	15.10±2.81def
NP1.7	8.70±2.63abc	13.20±2.10cdef	13.30±4.08ef	14.20±4.18efg
NP2.2	7.50±1.96bcd	12.70±1.49def	13.30±3.34ef	13.80±2.44efg
NP2.20	6.00±1.56d	11.90±2.73efg	13.30±1.64ef	13.50±1.58fg
NP2.23	7.20±1.32cd	11.70±1.83fg	15.60±2.32de	16.30±2.87cdef
NP2.24	9.80±1.69a	9.90±1.20g	11.10±2.28f	11.50±2.07g
NP3.4	9.30±1.42abc	14.40±1.51bcde	15.70±4.52de	16.80±3.33cde
NP3.13	7.30±1.25bcd	15.60±3.37bc	17.30±2.21bcd	17.70±2.16bcd
Average	7.91	13.06	15.94	16.67
Equality of Variances	5.383**	9.192**	8.045**	8.438**

Note: N=10, Different lowercase letters indicate significant differences (*p*<0.05), **indicates extremely significant differences (*p*<0.01)

Supplementary Table S4. Changes in Stem Base Diameter of Plants Across Different Months

NO.	July	August	September	October
P1.2	1.17±0.25efgh	1.52±0.29abc	1.55±0.14f	1.64±0.17fg
P1.8	1.65±0.36abc	1.80±0.21ab	2.19±0.31b	2.29±0.38b
P3.1	1.35±0.38de	1.83±0.19ab	2.00±0.21bcd	2.07±0.21cd
P3.3	1.08±0.13g	1.59±0.20bc	1.68±0.35ef	1.74±0.21efg
P3.7	1.03±0.20g	1.19±0.16d	1.85±0.28def	1.94±0.32cde
P3.8	1.42±0.20cd	1.63±0.29abc	1.89±0.25cde	1.96±0.18cde
P4.2	1.71±0.24ab	1.78±0.15a	2.21±0.27bc	2.28±0.24b
NP1.11	1.36±0.22de	1.64±0.32abc	1.79±0.08def	1.85±0.10defg
NP1.12	1.09±0.20fg	1.58±0.19bc	1.85±0.17def	1.92±0.18cde
NP1.24	1.51±0.20bcd	1.56±0.18bc	1.58±0.28ef	1.62±0.17g
NP1.7	1.18±0.23efg	1.46±0.13c	1.73±0.18ef	1.76±0.19efg
NP2.2	1.56±0.14bcd	1.57±0.20bc	1.90±0.17cde	2.06±0.18cd
NP2.20	1.62±0.22abc	1.62±0.27abc	2.04±0.24bcd	2.10±0.23bc
NP2.23	1.35±0.24de	1.70±0.21abc	1.82±0.29def	2.05±0.21cd
NP2.24	1.33±0.37def	1.53±0.46abc	1.57±0.49ef	1.63±0.18g

NP3.4	1.09±0.26fg	1.49±0.23c	1.79±0.26ef	1.86±0.18def
NP3.13	1.82±0.13a	1.91±0.29ab	2.94±0.52a	2.93±0.35a
Average	1.37	1.61	1.90	1.99
Equality of Variances	10.099**	3.771**	11.203**	22.595**

Note: N=10, Different lowercase letters indicate significant differences (*p*<0.05), **indicates extremely significant differences (*p*<0.01)

Supplementary Table S5. Comparison of Leaf Characteristics Among *Atriplex canescens* genotypes

NO.	Leaf Length	Maximum Leaf Width	Leaf Area	Shape Coefficient
P1.2	4.71±0.52efg	0.48±0.08i	1.51±0.23ef	0.68±0.08bcde
P1.8	3.86±0.33h	0.62±0.04cde	1.69±0.21e	0.71±0.025ab
P3.1	6.42±0.3a	0.61±0.04def	2.64±0.29b	0.68±0.02abcde
P3.3	4.89±0.48cdef	0.55±0.04hi	1.91±0.27d	0.71±0.03a
P3.7	4.72±0.53efg	0.64±0.06bcde	2.06±0.25cd	0.69±0.02abcd
P3.8	4.44±0.35g	0.56±0.04ghi	1.69±0.22e	0.68±0.02abcde
P4.2	3.46±0.18ij	0.60±0.04defg	1.47±0.11f	0.70±0.02abc
NP1.7	4.93±0.36cde	0.67±0.08b	2.19±0.28c	0.66±0.04def
NP1.11	4.86±0.46cdef	0.63±0.03bcde	1.92±0.24d	0.63±0.03f
NP1.12	3.72±0.28hi	0.59±0.02efgh	1.56±0.14ef	0.71±0.03ab
NP1.24	3.31±0.25j	0.61±0.06cdef	1.36±0.14f	0.67±0.03cde
NP2.2	4.81±0.32defg	0.65±0.04bcd	2.06±0.21cd	0.66±0.02def
NP2.20	5.21±0.43c	0.54±0.05h	1.91±0.22d	0.68±0.03abcde
NP2.23	6.37±0.42a	0.66±0.03bc	2.74±0.24b	0.65±0.03ef
NP2.24	5.13±0.31cd	0.57±0.06fghi	1.99±0.24cd	0.69±0.05abcde
NP3.4	4.51±0.34fg	0.61±0.06cdef	1.91±0.23d	0.70±0.03abcd
NP3.13	5.69±0.32b	0.75±0.05a	2.96±0.22a	0.69±0.02abcd
Average	4.77	0.61	1.97	0.68
Equality of Variances	54.183**	14.879**	39.906**	4.141**

Note: N=10, Different lowercase letters indicate significant differences (*p*<0.05), **indicates extremely significant differences (*p*<0.01)

Supplementary Table S6. Comparison of Chlorophyll Fluorescence Parameters Among *Atriplex canescens* strains

NO.	Fm	Fv/Fm	Y(II)	Y(NPQ)	qP
P1.2	0.1549±0.0139b	0.6790±0.0078a	0.4877±0.0223bc	0.2793±0.0268f	0.7170±0.0262cde
P1.8	0.1073±0.0081f	0.6277±0.0054bcd	0.5108±0.0213b	0.2953±0.0203ef	0.8121±0.0330a
P3.1	0.1266±0.0066de	0.6096±0.0063def	0.4648±0.0051cde	0.3400±0.0075cd	0.7624±0.0080abc
P3.3	0.1195±0.0056ef	0.6044±0.0042def	0.4233±0.0077f	0.3683±0.0066b	0.7003±0.0136de
P3.7	0.1536±0.0042b	0.6494±0.0071b	0.5161±0.0065b	0.2882±0.0071f	0.7954±0.0086ab
P3.8	0.1546±0.0089b	0.6445±0.0124bc	0.4816±0.0377bc	0.2990±0.0173ef	0.7497±0.0732bcd
P4.2	0.1316±0.0038de	0.6367±0.0015bc	0.4820±0.0059bc	0.3159±0.0040de	0.7572±0.0108abc

NP1.11	0.1227±0.0006e	0.5972±0.0092f	0.4121±0.0226f	0.3727±0.0152a	0.6901±0.0371e
NP1.12	0.1375±0.0052cd	0.5632±0.0368g	0.3537±0.0347g	0.2821±0.0044f	0.6343±0.0473f
NP1.24	0.1261±0.0024de	0.6101±0.0009def	0.4380±0.0017ef	0.3464±0.0011bc	0.7175±0.0039cde
NP1.7	0.1739±0.009a	0.6782±0.0132a	0.5249±0.0051a	0.2547±0.0048g	0.7747±0.0226ab
NP2.2	0.1558±0.0023b	0.6510±0.0218b	0.4108±0.0138f	0.3143±0.0229de	0.6322±0.0013f
NP2.20	0.1210±0.0147ef	0.6212±0.0051cde	0.4398±0.0166def	0.3456±0.0132bc	0.7094±0.0298cde
NP2.23	0.1526±0.0116b	0.6474±0.0110b	0.4884±0.0195bc	0.2885±0.0242f	0.7582±0.0185abc
NP2.24	0.1458±0.0044bc	0.6364±0.0022bc	0.4716±0.0053cd	0.3272±0.0067cd	0.7404±0.0079bcde
NP3.13	0.1529±0.0002121b	0.6455±0.0015bc	0.4920±0.0011bc	0.3191±0.0004de	0.7637±0.0021abc
NP3.4	0.1510±0.0028bc	0.6003±0.0072ef	0.4188±0.0061f	0.3731±0.0069a	0.6977±0.0131de

Supplementary Table S7. Weighting of Factors Affecting *Atriplex canescens* Growth Calculated Using Entropy Weighting Method

Indicator	Information Entropy Value e	Information Utility Value d	Weighting Coefficient w
**Plant height**	0.9239	0.0761	9.01%
**Branch Number**	0.9246	0.0754	8.93%
**Node Number**	0.9446	0.0554	6.56%
**Stem Base Diameter**	0.8753	0.1247	14.76%
**Leaf Area**	0.9075	0.0925	10.95%
**Leaf Length**	0.9275	0.0725	8.58%
**Maximum Leaf Width**	0.9553	0.0447	5.30%
**Shape Coefficient**	0.9584	0.0416	4.93%
**Fm**	0.9417	0.0583	6.90%
**Fv/Fm**	0.9571	0.0429	5.08%
**Y(II)**	0.9592	0.0408	4.83%
**Y(NPQ)**	0.9398	0.0602	7.12%
**qP**	0.9403	0.0597	7.07%

Supplementary Table S8. TOPSIS-Based Comprehensive Evaluation Ranking of *Atriplex canescens* genotypes

strains	Positive Ideal Distance(D^+)	Negative Ideal Distance(D^-)	Relative Proximity(Ci)	Rank
P1.2	0.2213	0.1264	0.3634	14
P1.8	0.1839	0.1549	0.4572	7
P3.1	0.1325	0.1943	0.5945	2
P3.3	0.1939	0.1372	0.4144	10
P3.7	0.1616	0.1579	0.4943	5
P3.8	0.1749	0.1446	0.4525	8
P4.2	0.1910	0.1334	0.4111	11
NP1.11	0.1654	0.1839	0.5265	4
NP1.12	0.1946	0.1233	0.3878	12
NP1.24	0.2530	0.0820	0.2448	17

NP1.7	0.2337	0.0991	0.2979	16
NP2.2	0.1935	0.1176	0.3781	13
NP2.20	0.1665	0.1462	0.4674	6
NP2.23	0.1408	0.1910	0.5756	3
NP2.24	0.2260	0.1236	0.3536	15
NP3.13	0.0689	0.2533	0.7862	1
NP3.4	0.1843	0.1386	0.4292	9

Supplementary Table S9. Base Sequence Characteristics of *Atriplex canescens* genotypes

NO.	Base Content				Sequence Length
	A	T	G	C	
P1.2	19.03%	22.53%	29.07%	29.38%	657
P1.8	18.81%	22.48%	29.20%	29.51%	654
P2.22	18.87%	22.53%	29.22%	29.38%	657
P3.1	18.81%	22.63%	29.20%	29.36%	654
P3.18	19.03%	22.53%	29.07%	29.22%	657
P3.3	19.18%	22.37%	28.92%	29.38%	657
P3.7	18.81%	22.63%	29.36%	29.20%	654
P3.8	18.65%	22.78%	29.20%	29.36%	654
P4.13	19.79%	22.37%	28.61%	29.22%	657
P4.2	19.79%	22.22%	28.61%	29.38%	657
P4.4	19.63%	22.37%	28.61%	29.38%	657
P5.15	18.72%	22.68%	29.22%	29.38%	657
P7.18	18.65%	22.78%	29.20%	29.36%	654
P7.19	18.87%	22.68%	29.22%	29.22%	657
P7.7	19.79%	22.53%	28.46%	29.22%	657
P9.17	19.79%	22.37%	28.77%	29.07%	657
P9.18	19.63%	22.22%	28.92%	29.22%	657
P10.14	19.79%	22.37%	28.61%	29.22%	657
NP1.10	18.65%	22.78%	29.20%	29.36%	654
NP1.11	19.42%	22.48%	28.90%	29.05%	654
NP1.12	18.87%	22.22%	29.38%	29.38%	657
NP1.24	18.87%	22.53%	29.22%	29.38%	657
NP1.6	19.18%	22.37%	29.07%	29.38%	657
NP1.7	18.65%	22.94%	29.05%	29.36%	654
NP2.14	18.81%	22.63%	29.20%	29.36%	654

NP2.2	18.96%	22.48%	29.20%	29.36%	654
NP2.20	18.87%	22.37%	29.22%	29.38%	657
NP2.23	18.72%	22.68%	29.22%	29.38%	657
NP2.24	19.57%	22.63%	28.59%	29.20%	654
NP3.13	18.81%	22.63%	29.20%	29.36%	654
NP3.4	18.65%	22.78%	29.20%	29.36%	654
Average	19.09%	22.54%	29.04%	29.32%	655.74

Supplementary Table S10. Genetic Distances Among Different Clones of *Atriplex canescens* genotypes

NO.	P1.	P	P2.	P3.	P3.	P3.	P3.	P3.	P4.	P4.	P4.	P5.	P7.	P7.	P7.	P9.	P9.	P10	NP	NP	NP	NP	NP	NP	NP	NP	NP	NP	NP	NP
P1.8	0.0																													
P2.22	0.0	0																												
P3.1	0.0	0	0.0																											
P3.18	0.0	0	0.0	0.0																										
P3.3	0.0	0	0.0	0.0	0.0																									
P3.7	0.0	0	0.0	0.0	0.0	0.0																								
P3.8	0.0	0	0.0	0.0	0.0	0.0	0.0																							
P4.13	0.0	0	0.0	0.0	0.0	0.0	0.0	0.0																						
P4.2	0.0	0	0.0	0.0	0.0	0.0	0.0	0.0	0.0																					
P4.4	0.0	0	0.0	0.0	0.0	0.0	0.0	0.0	0.0	0.0																				
P5.15	0.0	0	0.0	0.0	0.0	0.0	0.0	0.0	0.0	0.0	0.0																			
P7.18	0.0	0	0.0	0.0	0.0	0.0	0.0	0.0	0.0	0.0	0.0	0.0																		
P7.19	0.0	0	0.0	0.0	0.0	0.0	0.0	0.0	0.0	0.0	0.0	0.0	0.0																	
P7.7	0.0	0	0.0	0.0	0.0	0.0	0.0	0.0	0.0	0.0	0.0	0.0	0.0	0.0																
P9.17	0.0	0	0.0	0.0	0.0	0.0	0.0	0.0	0.0	0.0	0.0	0.0	0.0	0.0	0.0															
P9.18	0.0	0	0.0	0.0	0.0	0.0	0.0	0.0	0.0	0.0	0.0	0.0	0.0	0.0	0.0	0.0														
P10.1	0.0	0	0.0	0.0	0.0	0.0	0.0	0.0	0.0	0.0	0.0	0.0	0.0	0.0	0.0	0.0	0.0													
NP1.1	0.0	0	0.0	0.0	0.0	0.0	0.0	0.0	0.0	0.0	0.0	0.0	0.0	0.0	0.0	0.0	0.0	0.0												
NP1.1	0.0	0	0.0	0.0	0.0	0.0	0.0	0.0	0.0	0.0	0.0	0.0	0.0	0.0	0.0	0.0	0.0	0.0	0.0											
NP1.1	0.0	0	0.0	0.0	0.0	0.0	0.0	0.0	0.0	0.0	0.0	0.0	0.0	0.0	0.0	0.0	0.0	0.0	0.0	0.0										
NP1.2	0.0	0	0.0	0.0	0.0	0.0	0.0	0.0	0.0	0.0	0.0	0.0	0.0	0.0	0.0	0.0	0.0	0.0	0.0	0.0	0.0									
NP1.6	0.0	0	0.0	0.0	0.0	0.0	0.0	0.0	0.0	0.0	0.0	0.0	0.0	0.0	0.0	0.0	0.0	0.0	0.0	0.0	0.0	0.0								
NP1.7	0.0	0	0.0	0.0	0.0	0.0	0.0	0.0	0.0	0.0	0.0	0.0	0.0	0.0	0.0	0.0	0.0	0.0	0.0	0.0	0.0	0.0	0.0							
NP2.1	0.0	0	0.0	0.0	0.0	0.0	0.0	0.0	0.0	0.0	0.0	0.0	0.0	0.0	0.0	0.0	0.0	0.0	0.0	0.0	0.0	0.0	0.0	0.0						
NP2.2	0.0	0	0.0	0.0	0.0	0.0	0.0	0.0	0.0	0.0	0.0	0.0	0.0	0.0	0.0	0.0	0.0	0.0	0.0	0.0	0.0	0.0	0.0	0.0	0.0					
NP2.2	0.0	0	0.0	0.0	0.0	0.0	0.0	0.0	0.0	0.0	0.0	0.0	0.0	0.0	0.0	0.0	0.0	0.0	0.0	0.0	0.0	0.0	0.0	0.0	0.0	0.0				
NP2.2	0.0	0	0.0	0.0	0.0	0.0	0.0	0.0	0.0	0.0	0.0	0.0	0.0	0.0	0.0	0.0	0.0	0.0	0.0	0.0	0.0	0.0	0.0	0.0	0.0	0.0	0.0			
NP2.2	0.0	0	0.0	0.0	0.0	0.0	0.0	0.0	0.0	0.0	0.0	0.0	0.0	0.0	0.0	0.0	0.0	0.0	0.0	0.0	0.0	0.0	0.0	0.0	0.0	0.0	0.0	0.0		
NP3.1	0.0	0	0.0	0.0	0.0	0.0	0.0	0.0	0.0	0.0	0.0	0.0	0.0	0.0	0.0	0.0	0.0	0.0	0.0	0.0	0.0	0.0	0.0	0.0	0.0	0.0	0.0	0.0	0.0	
NP3.4	0.0	0	0.0	0.0	0.0	0.0	0.0	0.0	0.0	0.0	0.0	0.0	0.0	0.0	0.0	0.0	0.0	0.0	0.0	0.0	0.0	0.0	0.0	0.0	0.0	0.0	0.0	0.0	0.0	0.0

Supplementary Table S11. Analysis of Genetic Diversity Among Different genotypes

NO.	Base Deletions	Conserved Sites	Polymorphic Sites	h	Hd	Pi
P1.2	18	651	6	2	0.667±0.204	0.006 ±0.002
P1.8	21	171	483	2	0.667±0.204	0.492 ±0.151
P2.22	18	657	0	1	0.000±0.000	0.000 ±0.000
P3.1	21	170	484	2	0.667±0.204	0.493 ±0.151
P3.18	21	168	486	2	0.667±0.204	0.495 ±0.152
P3.3	18	653	4	3	0.833±0.222	0.004 ±0.001
P3.7	36	196	443	3	0.833±0.222	0.462 ±0.141
P3.8	21	170	484	2	0.667±0.204	0.493 ±0.151
P4.13	18	645	12	2	0.667±0.204	0.012 ±0.004
P4.2	18	646	11	2	0.667±0.204	0.011 ±0.003
P4.4	36	196	443	2	0.667±0.204	0.462 ±0.142
P5.15	36	196	443	2	0.667±0.204	0.462 ±0.142
P7.18	21	168	486	2	0.667±0.204	0.495 ±0.152
P7.19	36	197	442	2	0.667±0.204	0.461 ±0.141
P7.7	18	614	43	2	0.667±0.204	0.044 ±0.013
P9.17	18	644	13	2	0.667±0.204	0.013 ±0.004
P9.18	18	644	13	2	0.667±0.204	0.013 ±0.004
P10.14	18	645	12	2	0.667±0.204	0.012 ±0.004
NP1.10	21	168	486	2	0.667±0.204	0.495 ±0.152
P1.11	21	168	486	3	0.833±0.222	0.496 ±0.152
P1.12	18	651	6	3	0.833±0.222	0.006 ±0.002
NP1.24	36	197	442	2	0.667±0.204	0.461 ±0.141
NP1.6	18	654	3	2	0.667±0.204	0.003 ±0.001
NP1.7	21	169	485	2	0.667±0.204	0.494 ±0.151
P2.14	21	170	484	2	0.667±0.204	0.493 ±0.151
NP2.2	21	169	485	2	0.667±0.204	0.494 ±0.151
NP2.20	18	602	55	3	0.833±0.222	0.056 ±0.017
P2.23	36	197	442	2	0.667±0.204	0.461 ±0.141
NP2.24	21	167	487	2	0.667±0.204	0.496 ±0.152
NP3.13	21	170	484	2	0.667±0.204	0.493 ±0.151
NP3.4	21	168	486	2	0.667±0.204	0.495 ±0.152

Note: **h**: Number of haplotypes; **Hd**: Haplotype diversity; **Pi**: Nucleotide Diversity

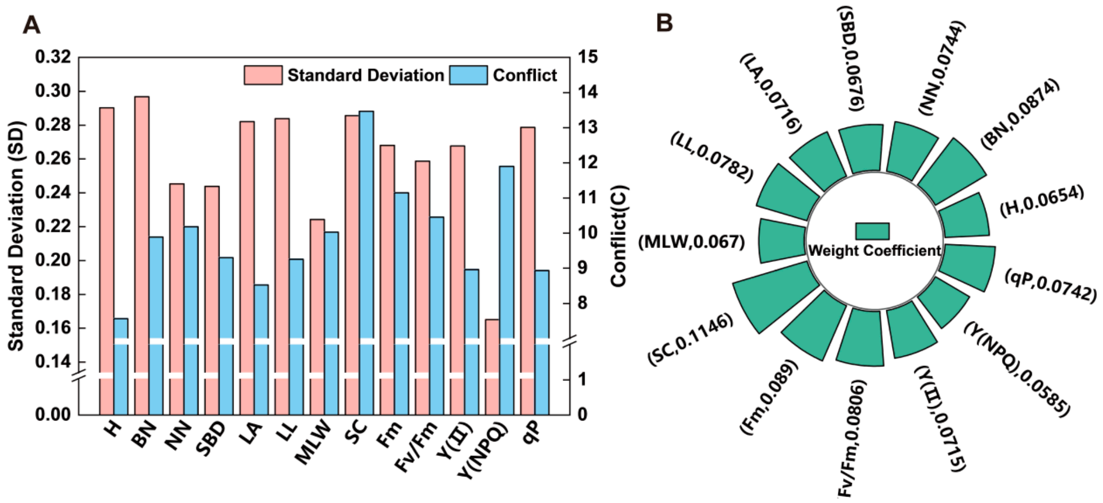

Supplementary Figure S1. CRITIC method Evaluation of 17 *A. canescens* genotypes. **(A)** Variability and conflict values of phenotypic traits, and physiological indicators. Variability was quantified by the normalized standard deviation; conflict was calculated as one minus the absolute value of the Pearson correlation coefficient between the target indicator and all other indicators; **(B)** weight coefficients of phenotypic traits, and physiological indicators calculated by CRITIC method .

Supplementary Table S12: the rankings of 17 *Atriplex canescens* genotypes under both methods

Genotypes	Ranking of TOPSIS method	Ranking of CRITIC method
P1.2	14	9
P1.8	7	8
P3.1	2	3
P3.3	10	11
P3.7	5	4
P3.8	8	6
P4.2	11	12
NP1.11	4	2
NP1.12	12	15
NP1.24	17	17
NP1.7	16	16
NP2.2	13	14
NP2.20	6	7
NP2.23	3	5
NP2.24	15	13
NP3.13	1	1
NP3.4	9	10
